# Mutant uromodulin expression leads to altered homeostasis of the endoplasmic reticulum and activates the unfolded protein response

**DOI:** 10.1371/journal.pone.0175970

**Published:** 2017-04-24

**Authors:** Céline Schaeffer, Stefania Merella, Elena Pasqualetto, Dejan Lazarevic, Luca Rampoldi

**Affiliations:** 1 Molecular Genetics of Renal Disorders, Division of Genetics and Cell Biology, IRCCS San Raffaele Scientific Institute, Milan, Italy; 2 Center of Translational Genomics and Bioinformatics, IRCCS San Raffaele Scientific Institute, Milan, Italy; Duke University School of Medicine, UNITED STATES

## Abstract

Uromodulin is the most abundant urinary protein in physiological conditions. It is exclusively produced by renal epithelial cells lining the thick ascending limb of Henle’s loop (TAL) and it plays key roles in kidney function and disease. Mutations in *UMOD*, the gene encoding uromodulin, cause autosomal dominant tubulointerstitial kidney disease uromodulin-related (ADTKD-*UMOD*), characterised by hyperuricemia, gout and progressive loss of renal function. While the primary effect of *UMOD* mutations, retention in the endoplasmic reticulum (ER), is well established, its downstream effects are still largely unknown. To gain insight into ADTKD-*UMOD* pathogenesis, we performed transcriptional profiling and biochemical characterisation of cellular models (immortalised mouse TAL cells) of robust expression of wild type or mutant GFP-tagged uromodulin. In this model mutant uromodulin accumulation in the ER does not impact on cell viability and proliferation. Transcriptional profiling identified 109 genes that are differentially expressed in mutant cells relative to wild type ones. Up-regulated genes include several ER resident chaperones and protein disulphide isomerases. Consistently, pathway enrichment analysis indicates that mutant uromodulin expression affects ER function and protein homeostasis. Interestingly, mutant uromodulin expression induces the Unfolded Protein Response (UPR), and specifically the IRE1 branch, as shown by an increased splicing of XBP1. Consistent with UPR induction, we show increased interaction of mutant uromodulin with ER chaperones Bip, calnexin and PDI. Using metabolic labelling, we also demonstrate that while autophagy plays no role, mutant protein is partially degraded by the proteasome through ER-associated degradation. Our work demonstrates that ER stress could play a central role in ADTKD-*UMOD* pathogenesis. This sets the bases for future work to develop novel therapeutic strategies through modulation of ER homeostasis and associated protein degradation pathways.

## Introduction

Mutations in the *UMOD* gene, encoding for uromodulin, also known as Tamm-Horsfall protein, are responsible for a rare autosomal dominant form of tubulointerstitial kidney disease referred as ADTKD-*UMOD* [1]. ADTKD-*UMOD* (MIM 162000, 603860, 191845) has an estimated prevalence of 1:100.000 (www.orpha.net). It shares some common features with autosomal dominant tubulointerstitial kidney diseases caused by mutations in *MUC1* (mucin 1, 1q21) [2], *HNF1B* (HNF1beta, 17q12) [3], *REN* (renin, 1q32) [4] and *SEC61A1* (Sec 61 translocon alpha 1 subunit, 3q21) [5]. While all forms of ADTKD present with interstitial fibrosis, tubular atrophy and dilation, and thickening and lamellation of tubular basal membranes, ADTKD-*UMOD* is typically characterised by decreased fractional excretion of urate, causing hyperuricaemia and often gout [1]. ADTKD-*UMOD* is heterogeneous in several clinical aspects, including clinical appearance, age at onset, presence of cysts, and rate of progression to end-stage renal disease. No specific therapy is currently available, other than renal replacement therapy.

Uromodulin is a 105 kDa glycosylphosphatidylinositol (GPI)-anchored protein specifically produced by epithelial cells lining the thick ascending limb of Henle’s loop (TAL) and released into the urine after cleavage by the protease hepsin [6,7]. It is the most abundant protein in urine in physiological conditions where it is present as high-molecular-weight filamentous polymers. The biological function of uromodulin is still not fully understood. Studies in *Umod* knock-out mice and recent evidence in patients with urinary tract infections or kidney stones showed that urinary uromodulin has a protective role against these conditions [8–11]. Moreover, it was shown to regulate sodium absorbance in the TAL [12] and proposed to act as a modulator of renal innate immunity, acting as a damage-associated molecular pattern that can activate interstitial dendritic cells when released in the interstitium [13], and as a protective factor for renal tubules after acute kidney injury [14,15].

To date over 100 *UMOD* mutations have been described. All but 4 (in-frame deletions) are missense changes. We and others demonstrated that *UMOD* mutations have a clear common effect, as they lead to defective trafficking to the plasma membrane and endoplasmic reticulum (ER) retention of mutant uromodulin [6], pointing at this disease as an additional member of ER storage diseases [16]. This is consistent with findings in patient renal biopsies, typically showing the presence of large intracellular aggregates of uromodulin in TAL epithelial cells and abnormal expansion of ER cisternae [17,18], and dramatic reduction of uromodulin levels in patient urines [17].

While the primary effect of *UMOD* mutations, i.e. retention in the ER, is well established, its downstream effects are still largely uncharacterised. Studies on ADTKD-*UMOD* mouse models that recapitulate the main features of the human disease show induction of inflammatory responses [19,20] and of the non-canonical NFkB pathway in the TAL [21]. However, no high-throughput study has been carried out at the cellular level in order to identify dysregulated pathways upon mutant uromodulin expression. To this goal, we performed transcriptional profiling and biochemical characterisation of cellular models of robust expression of wild type or mutant GFP-tagged uromodulin isoforms.

## Results

### mTAL cells as a cellular model of mutant uromodulin expression

**Fig 1:**
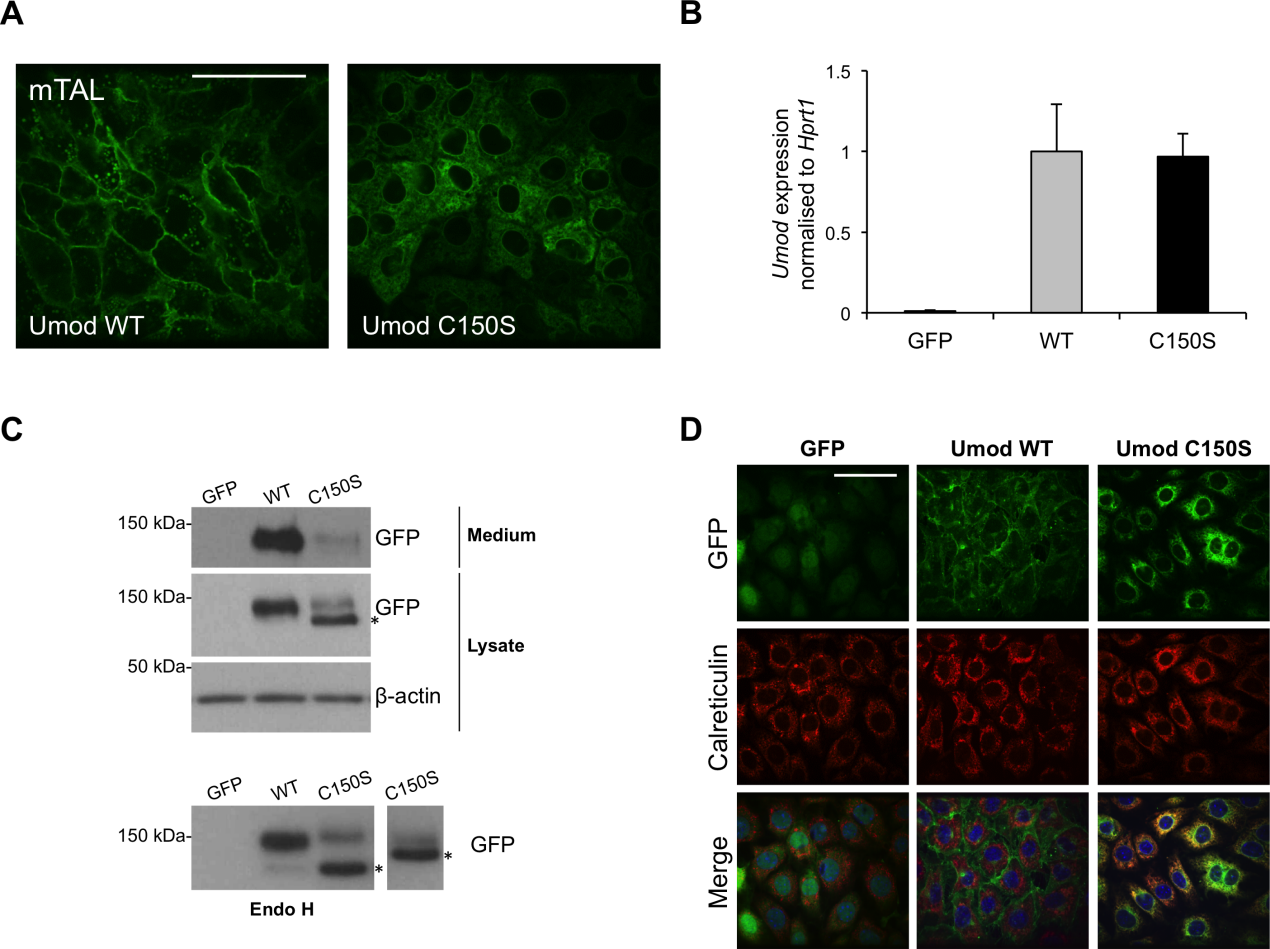
Characterisation of mTAL cells expressing wild type or mutant uromodulin isoforms. (A) Live imaging showing GFP signal in mTAL cells expressing GFP-tagged WT or C150S mutant uromodulin isoform. Bar = 40 μm. (B) Uromodulin expression assessed by real-time RT-qPCR. Expression is normalised to *Hprt1*. Cells expressing GFP alone are shown as negative control (n = 5 independent experiments) (C) Western-blot analysis of mTAL cells expressing WT or C150S mutant uromodulin isoform. * indicates the ER glycosylated form that is Endo H sensitive (see panel below). (D) Immunofluorescence analysis of mTAL cells expressing GFP-tagged uromodulin isoforms. GFP signal is shown in green. Calreticulin, used as an ER marker, is shown in red. Merged pictures show ER localisation of mutant uromodulin while the wild type protein is trafficked to the membrane. Bar = 40 μm.

To gain insight into the molecular pathways activated by mutant uromodulin expression, we generated a new cellular model of uromodulin expression using immortalized epithelial cells isolated from murine TAL segments (mTAL). Although this method has been described to keep cells in a differentiated state [22,23], we did not observe expression of specific TAL markers, as uromodulin or NKCC2, after immortalization. Thus, uromodulin expression was obtained by transduction with a lentiviral construct coding for wild type or mutant (C150S) uromodulin isoforms fused to GFP. In order to reach high expression level, comparable to the one observed *in vivo* in TAL cells (see below), *UMOD* expression was placed under the control of the spleen focus-forming virus promoter. As a paradigm mutation, we expressed patient mutation C150S that has been extensively characterised in other cell models [24,25]. Live imaging of transduced cells shows clear membrane enrichment for wild type uromodulin and intracellular, reticular distribution of the C150S mutated protein (Fig 1A). The expression level of uromodulin in the two cell lines is comparable, as determined by quantitative real-time RT-PCR (Fig 1B). Western blot analysis shows enrichment of an Endo H sensitive form, corresponding to the high-mannose ER precursor, in cells expressing C150S uromodulin (Fig 1C). Consistently, mutant isoform shows reduced secretion in cell culturing medium. ER retention of C150S uromodulin was confirmed by its increased co-localisation with the ER marker calreticulin through immunofluorescence analysis (Fig 1D). Thus, mutant uromodulin shows delayed trafficking to the plasma membrane and accumulation in the ER in mTAL cells, as we previously reported for MDCK and HEK293 cells [24,25].

**Fig 2:**
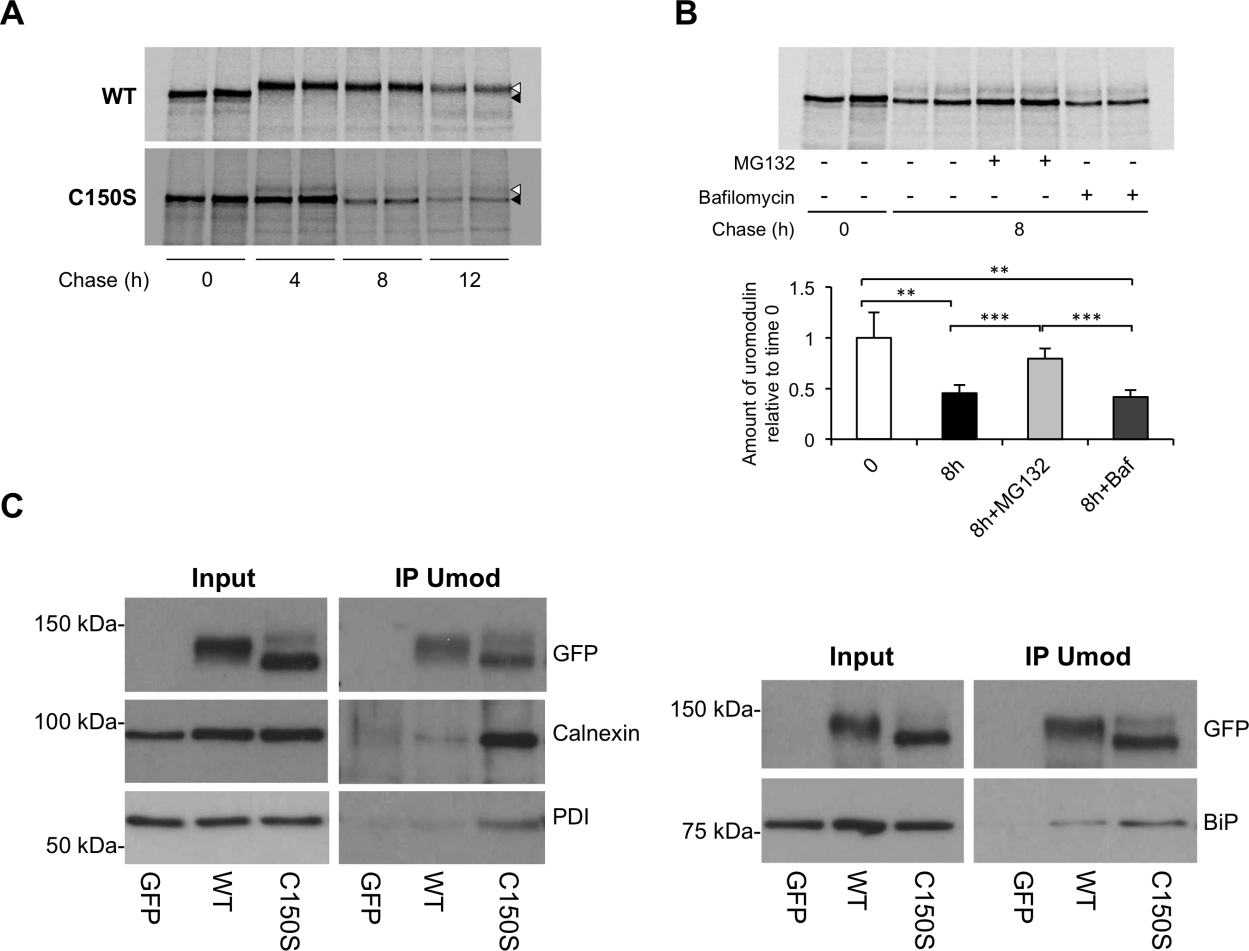
Stability of wild type and C150S mutant uromodulin in mTAL cells. (A) Pulse-chase experiment showing maturation of wild type and C150S uromodulin isoforms in mTAL cells. Wild type uromodulin is completely matured into the Golgi-type glycosylated form (white arrow) after 4 hours of chase, while the mutant one shows mainly the ER-type glycosylated form (black arrow) even after 12 hours. (B) Pulse-chase experiment showing mutant uromodulin stability in presence of proteasome (MG132) or autophagy (bafilomycin) inhibitors. Mutant uromodulin is stabilised by treatment with MG132 suggesting the involvement of the proteasome for its degradation. *P<0.05, **P<0.01, ***P<0.005 (Student t test) (n = 4 independent experiments). (C) Western blot analysis showing increased co-immunoprecipitation of ER chaperones calnexin, PDI and BiP with mutant uromodulin relative to wild type one.

We next assessed uromodulin stability and degradation through pulse-chase experiments. After 4 hours of chase wild type uromodulin reaches the mature, Golgi-type glycosylation state, while the mutant protein keeps immature, ER-type glycosylation (Fig 2A). After 8 hours of chase, the ER form of the mutant is clearly decreased compared to time 0 without a clear increase of the mature form, suggesting that most of mutant protein is intracellularly degraded. Mutant protein does not show maturation even after 12 hours of chase, but rather a further decrease of its ER form, while wild type protein is present only in its mature form. The slight decrease observed in the amount of mature wild type uromodulin at this time point is likely due to its secretion in the cellular medium. In cells expressing mutant uromodulin we then carried out an 8-hours chase in the presence of proteasome (MG132) or autophagy (bafilomycin) inhibitors. While MG132 treatment stabilises uromodulin, bafilomycin treatment has no effect (Fig 2B). Stabilisation of uromodulin after block of proteasomal degradation was confirmed by treatment with a different inhibitor, i.e lactacystin (data not shown). These results strongly suggest that mutant uromodulin is degraded via the proteasome, possibly through the ER-associated degradation (ERAD) pathway. Through co-immunoprecipitation experiments followed by western blot analysis we identified BiP, calnexin and protein disulphide isomerase (PDI) as mutant uromodulin interactors (Fig 2C), suggesting the involvement of these chaperones in uromodulin folding.

### Transcriptional profiling of mTAL cells expressing wild type or mutant uromodulin

**Fig 3:**
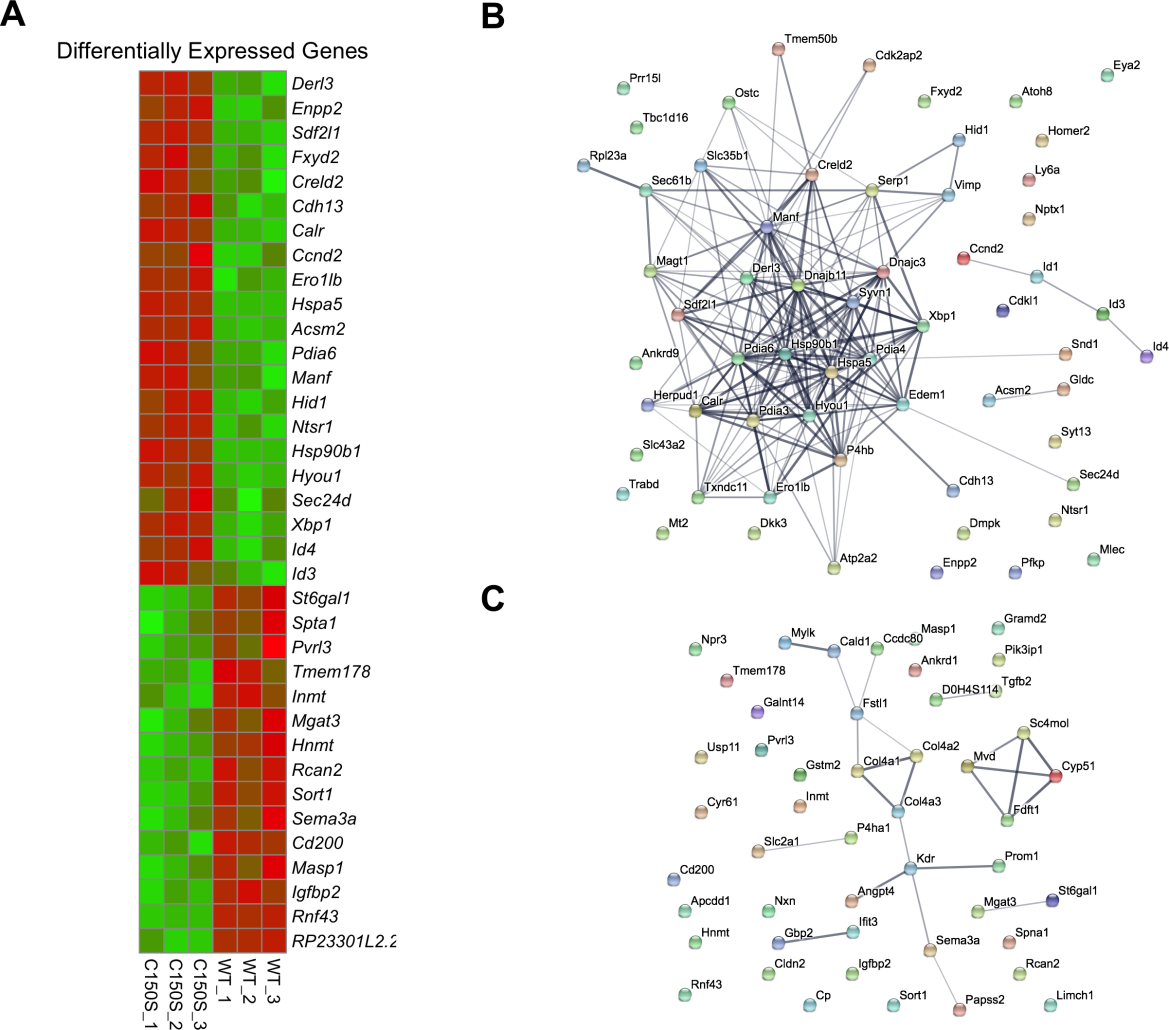
Transcriptome analysis in mTAL cells expressing wild type or C150S mutant uromodulin. (A) Heat map showing differentially expressed genes in mTAL cells expressing C150S uromodulin compared to wild type ones. Cut off: fold change > 1.5; P adjusted < 0.05. (B) STRING analysis showing networks formed by proteins encoded by up-regulated genes in mTAL cells expressing C150S uromodulin. Edges represent protein-protein association (physical or functional); their thickness is proportional to confidence. (C) Same analysis as in panel B for down-regulated genes. The proteins encoded by these genes are not forming relevant networks.

In order to identify the cellular processes affected by mutant uromodulin expression, we characterised the transcriptome of mTAL cells expressing wild type or C150S uromodulin isoforms by RNA sequencing. Obtained data have been deposited in NCBI Gene Expression Omnibus [26] and are accessible through GEO Series accession number GSE92704 (https://www.ncbi.nlm.nih.gov/geo/query/acc.cgi?acc=GSE92704). Among the 12,346 expressed genes, we identified a total of 109 genes differentially expressed (adjusted P<0.05) in mutant expressing cells compared to wild type, including 21 genes up-regulated and 14 down-regulated with a fold change >1.5 (Fig 3A). Interestingly, the *UMOD* transcript represented about 0.5% of the total transcriptome in both cell lines, comparable with what reported for endogenous *UMOD* in RNA sequencing analysis of microdissected rat medullary TALs (0.8%) [27]. Analysis of protein interaction networks (including functional and physical interactions) using STRING software [28] shows extensive connection between genes up-regulated in mutant expressing cells (Fig 3B). Interestingly, the vast majority of interconnected proteins are ER or ER-related proteins. No relevant extended network can be seen for downregulated genes (Fig 3C).

Pathway enrichment analysis using Gene Set Enrichment Analysis (GSEA) [29,30] shows that mutant uromodulin expression strongly impacts on ER function and protein homeostasis (Table 1 and S1A Fig). Indeed, we could observe a significant induction of expression of genes involved in protein folding (e.g. *Hsp90*, *Pdia4*, *Pdia6*), Unfolded Protein Response (UPR) (e.g. *Hspa5*, *Xbp1*) and ERAD (e.g. *Derl3*, *Sdf2l1*). Consistent with the STRING outcome for downregulated genes, no pathway was significantly enriched in cells expressing wild type uromodulin.

**Table 1:** Upregulated pathways in mTAL cells expressing C150S uromodulin compared to wild type ones.

Pathways (GO Biological Process)	FDR*	Number of genes	Genes contributing to pathway enrichment
Cellular response to topologically incorrect protein	0	28/108 (122)	*Derl3*, *Sdf2l1*, *Calr*, *Hspa5*, *Pdia6*, *Hsp90b1*, *Hyou1*, *Xbp1*, *Dnajc3*, *Edem1*, *Syvn1*, *Sec61b*, *Dnajb11*, *Kdelr3*, *Serp1*, *Gfpt1*, *Herpud1*, *Sec31a*, *Rnf126*, *Creb3l2*, *Dnajb9*, *Derl1*, *Pdia5*, *Sec61a1*, *Eif2ak3*, *Wfs1*, *Sec61g*, *Atf6*
IRE1 mediated Unfolded Protein Response	0.001	18/54 (56)	*Hspa5*, *Pdia6*, *Hyou1*, *Xbp1*, *Dnajc3*, *Edem1*, *Syvn1*, *Sec61b*, *Dnajb11*, *Kdelr3*, *Serp1*, *Gfpt1*, *Sec31a*, *Dnajb9*, *Pdia5*, *Sec61a1*, *Wfs1*, *Sec61g*
Response to topologically incorrect protein	0.004	30/143 (163)	*Derl3*, *Sdf2l1*, *Calr*, *Hspa5*, *Pdia6*, *Manf*, *Hsp90b1*, *Hyou1*, *Xbp1*, *Dnajc3*, *Hspa1l*, *Edem1*, *Syvn1*, *Sec61b*, *Dnajb11*, *Kdelr3*, *Serp1*, *Gfpt1*, *Herpud1*, *Sec31a*, *Rnf126*, *Creb3l2*, *Dnajb9*, *Derl1*, *Pdia5*, *Sec61a1*, *Eif2ak3*, *Wfs1*, *Sec61g*, *Atf6*
ER associated ubiquitin dependent protein catabolic process	0.005	16/58 (61)	*Derl3*, *Sdf2l1*, *Hspa5*, *Hsp90b1*, *Edem1*, *Syvn1*, *Sec61b*, *Sel1l*, *Dnajb9*, *Derl1*, *Wfs1*, *Stt3b*, *Tmem129*, *Ubqln2*, *Fbxo2*, *Nploc4*
Negative regulation of response to endoplasmic reticulum stress	0.007	6/31 (39)	*Derl3*, *Hyou1*, *Xbp1*, *Dnajc3*, *Syvn1*, *Herpud1*
ERAD pathway	0.009	17/67 (74)	*Derl3*, *Sdf2l1*, *Hspa5*, *Hsp90b1*, *Edem1*, *Syvn1*, *Sec61b*, *Herpud1*, *Sel1l*, *Dnajb9*, *Derl1*, *Wfs1*, *Stt3b*, *Tmem129*, *Ubqln2*, *Fbxo2*, *Nploc4*
Response to endoplasmic reticulum stress	0.014	41/203 (233)	*Derl3*, *Sdf2l1*, *Calr*, *Hspa5*, *Pdia6*, *Hsp90b1*, *Hyou1*, *Xbp1*, *Pdia4*, *Dnajc3*, *Edem1*, *Syvn1*, *Txndc11*, *Pdia3*, *Sec61b*, *P4hb*, *Dnajb11*, *Kdelr3*, *Serp1*, *Gfpt1*, *Fam129a*, *Herpud1*, *Sec31a*, *Atp2a2*, *Sel1l*, *Txndc5*, *Anks4b*, *Uba5*, *Cebpb*, *Creb3l2*, *Dnajb9*, *Derl1*, *Pdia5*, *Sec61a1*, *Eif2ak3*, *Wfs1*, *Sec61g*, *Atf6*, *Nrbf2*, *Stt3b*, *Tmem129*
Protein exit from endoplasmic reticulum	0.021	11/17 (20)	*Hsp90b1*, *Syvn1*, *Sec61b*, *Herpud1*, *Sel1l*, *Surf4*, *Derl1*, *Lman1*, *Tmem129*, *Tmed9*, *Nploc4*

*FDR, False Discovery Rate. Number of genes, A/B (C): A, number of genes contributing to pathway enrichment; B, number of genes from the Gene Ontology (GO) pathway expressed in mTAL cells; C, total number of genes listed in the GO pathway.

Data obtained by RNA sequencing were validated by quantitative real-time RT-PCR (S1B Fig). We did not observe activation of pathways related to inflammation, oxidative stress or apoptosis. Absence of a toxic effect of mutant uromodulin expression on cell viability is also shown by the lack of expression of cleaved caspase 3 in C150S-expressing cells (S1C Fig).

### Activation of the IRE1 branch of the UPR in mTAL cells expressing mutant uromodulin

Since ER stress and UPR appeared to be the main, if not only, cellular response triggered by expression of mutant uromodulin, we thoroughly investigated UPR activation by assessing its three different sensors and effectors: IRE1 (inositol requiring enzyme 1), ATF6 (activating transcription factor 6) and PERK (double-stranded RNA-activated protein kinase (PKR)-like ER kinase) [31].

**Fig 4:**
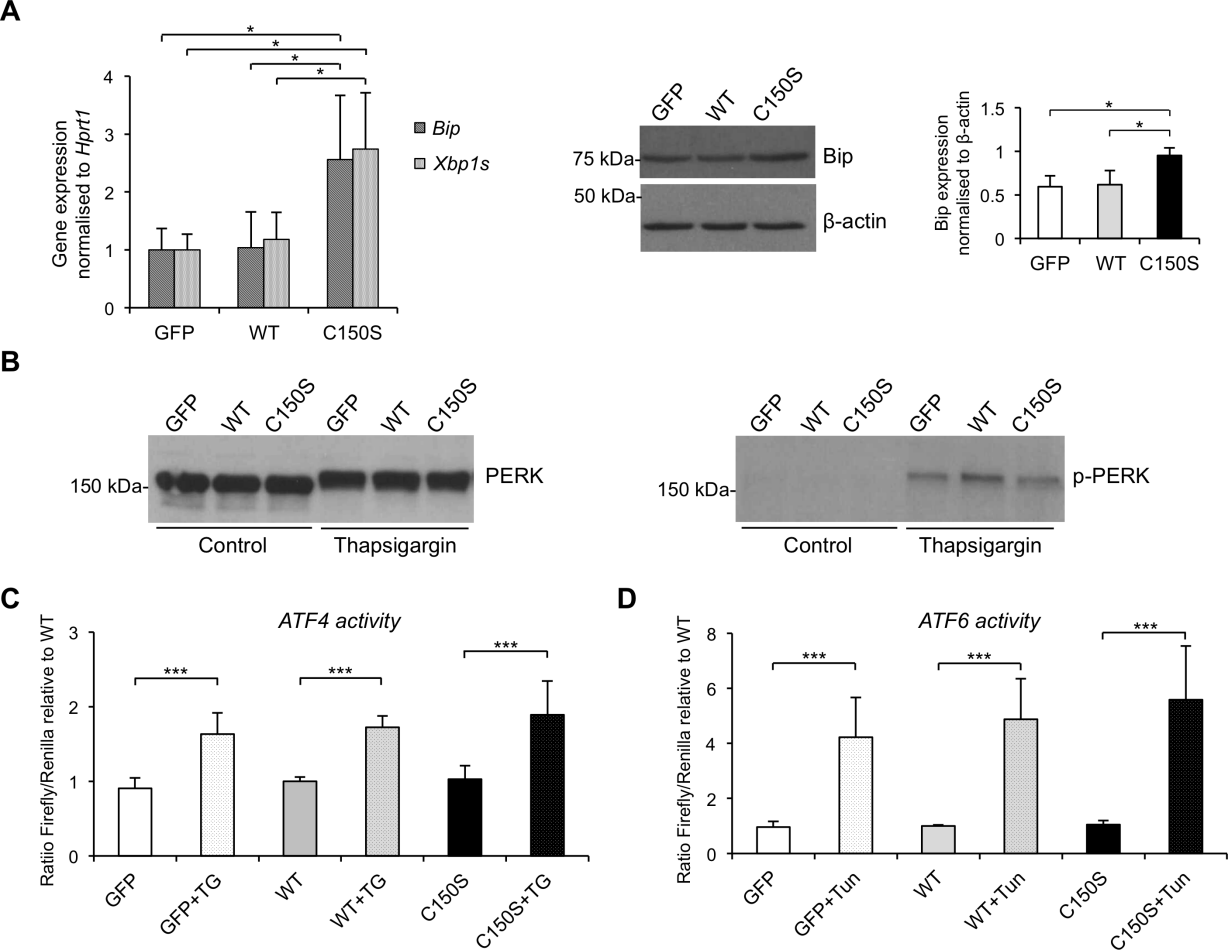
UPR induction in mTAL cells expressing wild type or C150S mutant uromodulin isoform. (A) *Bip* and spliced Xbp1 (*Xbp1s*) expression assessed by real-time RT-qPCR. Expression is normalised to *Hprt1* (n = 5 independent experiments) (left panel). Western blot analysis showing increased Bip protein levels in mTAL cells expressing mutant uromodulin (n = 3 independent experiments) (right panel). *P<0.05 (mutant *vs* wild type, Student t test). (B) Western blot analysis of PERK in mTAL cells expressing uromodulin at baseline and after incubation with tunicamycin (2 μg/mL for 14h). A shift in PERK migration is observed after treatment with tunicamycin, but not at baseline (left panel). Western blot performed with an antibody specific for the phosphorylated (Thr980) form of PERK shows the presence of the phosphorylated protein only in tunicamycin-treated cells (right panel). (C) ATF4 activity, assessed through the use of a luciferase-based, ATF4 reporter construct, is equally negligible in cells expressing wild type or C150S uromodulin, while it is evident in all cells upon thapsigargin treatment (100 nM for 14 h) ***P<0.001 (control *vs* thapsigargin, Student t test) (n = 6). (D) ATF6 activation assessed through the use of a luciferase-based, ATF6 reporter construct. No ATF6 activation is observed in mutant uromodulin expressing cells. Activation can be observed in all cell lines upon treatment with tunicamycin. ***P<0.005 (control *vs* tunicamycin, Student t test) (n = 8).

We first analysed *Bip* (Immunoglobulin heavy chain-binding protein) expression level through RT-qPCR (Fig 4A, left panel) and Western blot (Fig 4A, right panel) and detected increased expression of this chaperone in mutant expressing cells compared to both wild type uromodulin and GFP control cells, showing that mutant uromodulin expression induces ER stress.

IRE1 is a bifunctional ER transmembrane kinase/endoribonuclease. When activated, IRE1 performs unconventional splicing of the mRNA for the transcription factor XBP1 (X-box binding protein 1) that activates UPR target genes [32,33]. Expression level of spliced XBP1 (*Xbp1s*) was measured by RT-qPCR and, as observed for BiP, mutant expressing cells showed higher expression than wild type and control cells (Fig 4A), demonstrating activation of the IRE1 branch.

PERK is an ER-resident transmembrane kinase. When activated it phosphorylates itself and the ubiquitous translation initiation factor eIF2α, inhibiting mRNA translation and reducing protein load in the ER. However, some mRNAs containing short open reading frames in their 5’UTR, as the one encoding the transcription factor ATF4, are preferentially translated when eIF2 is inhibited, leading to increase levels of the encoded proteins [34]. We studied activation of the PERK branch of the UPR by assessing PERK phosphorylation and ATF4 protein level. Phosphorylation of PERK induces a shift in its electrophoretic mobility on SDS-PAGE [35] that is evident after tunicamycin treatment, but is not observed in mutant expressing cells (Fig 4B, left panel). Lack of increased PERK phosphorylation in cells expressing mutant uromodulin was confirmed by using an antibody specific for the phosphorylated form (Thr980) (Fig 4B, right panel). ATF4 protein levels were assessed by using a reporter construct in which the 5’UTR of the firefly luciferase gene is replaced by the one of ATF4 [34]. Consistent with the absence of PERK activation, no increase in luciferase activity could be observed in C150S uromodulin-expressing cells (Fig 4C).

ATF6 is a single-pass transmembrane protein that is cleaved proteolytically when unfolded proteins accumulate in the ER to release its N-terminal cytosolic domain that migrates into the nucleus acting as a transcription factor [36]. We assessed the activation of the ATF6 branch of the UPR through the use of a well-established reporter construct in which transcription of the firefly luciferase gene is regulated by the presence of five ATF6 binding sites upstream of the c-fos promoter [37]. While the reporter construct is properly activated when UPR is induced by tunicamycin treatment, no increase in luciferase activity can be observed in cells expressing mutant uromodulin, suggesting lack of activation of the ATF6 branch (Fig 4D).

All together, these results demonstrate that mutant uromodulin expression and accumulation in the ER induces the UPR through the IRE1 branch, with no apparent effect on the PERK and ATF6 arms. This is not due to any intrinsic property of mTAL cells that are able to mount a full UPR response upon treatment with chemical ER stressors (i.e. tunicamycin or thapsigargin). Notably, in such conditions maximal UPR was not different in wild type and mutant cells.

### Activation of the UPR is reproduced in MDCK cells expressing mutant uromodulin

To verify that the effect of mutant uromodulin expression on ER stress is not limited to mTAL cells, we generated MDCK cell lines transduced with lentiviral vectors for GFP-tagged wild-type or C150S mutant uromodulin. Similarly to what observed in mTAL cells, wild-type protein reaches the plasma membrane while the mutant one is intracellularly retained (S2A Fig). Analysis of mRNA expression level shows comparable expression of both uromodulin isoforms (S2B Fig). ER retention of mutant protein was demonstrated by western blot analysis, showing accumulation of the ER-type glycosylated form (S2C Fig), and by immunofluorescence analysis, showing increased co-localisation of the C150S isoform with the ER marker KDEL (S2D Fig).

**Fig 5:**
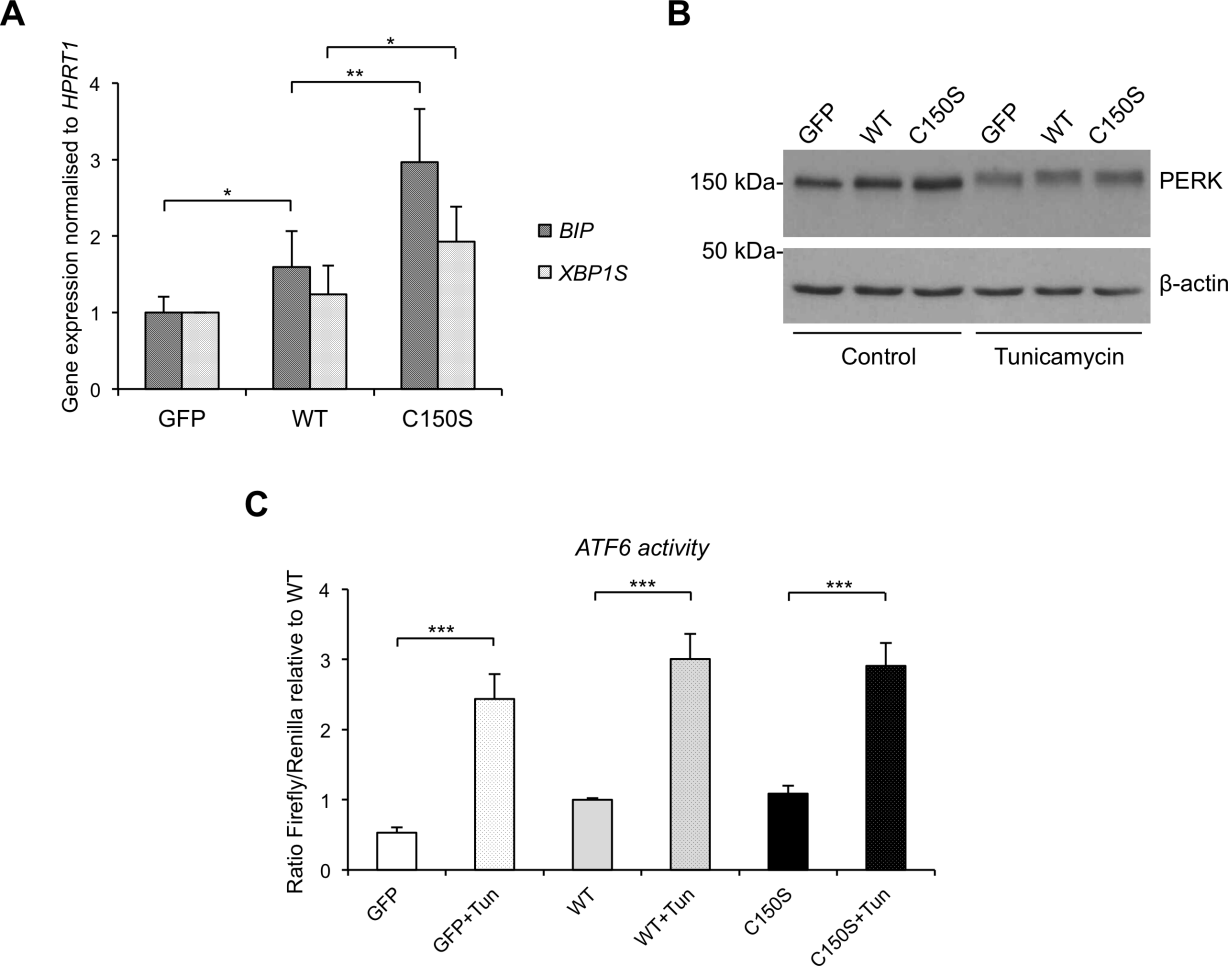
UPR induction in MDCK cells expressing wild type or C150S uromodulin isoforms. (A) *BIP* and *XBP1S* expression assessed by real-time RT-qPCR. Expression is normalised to *HPRT1*. *P<0.05, **P<0.01 (Student t test) (n = 5 independent experiments). (B) Western blot analysis of PERK in MDCK cells expressing wild type or C150S mutant uromodulin. A shift in PERK migration is seen upon tunicamycin treatment (2 μg/mL for 14 h), but not at baseline. (C) ATF6 activation assessed through the use of an ATF6 reporter construct. No ATF6 activation is observed in mutant uromodulin expressing cells. Activation can be observed in all cell lines upon tunicamycin treatment. ***P<0.005 (control *vs* tunicamycin-treated, Student t test) (n = 6 independent experiments).

Also in this cellular model mutant uromodulin expression induces ER stress as seen by increased expression of BiP (Fig 5A). We also confirmed increased activation of the IRE1 branch of the UPR through increased level of spliced XBP1 (Fig 5A). Activation of the other two UPR branches, i.e. PERK phosphorylation (Fig 5B) and ATF6-dependent transcriptional activity (Fig 5C), was similarly negligible in cells expressing wild type or mutant uromodulin. These data significantly strengthen findings in mTAL cells demonstrating that mutant uromodulin induces the UPR through activation of the IRE1 branch.

### UPR induction in cells expressing mild uromodulin mutants associated with ADTKD-*UMOD*

**Fig 6:**
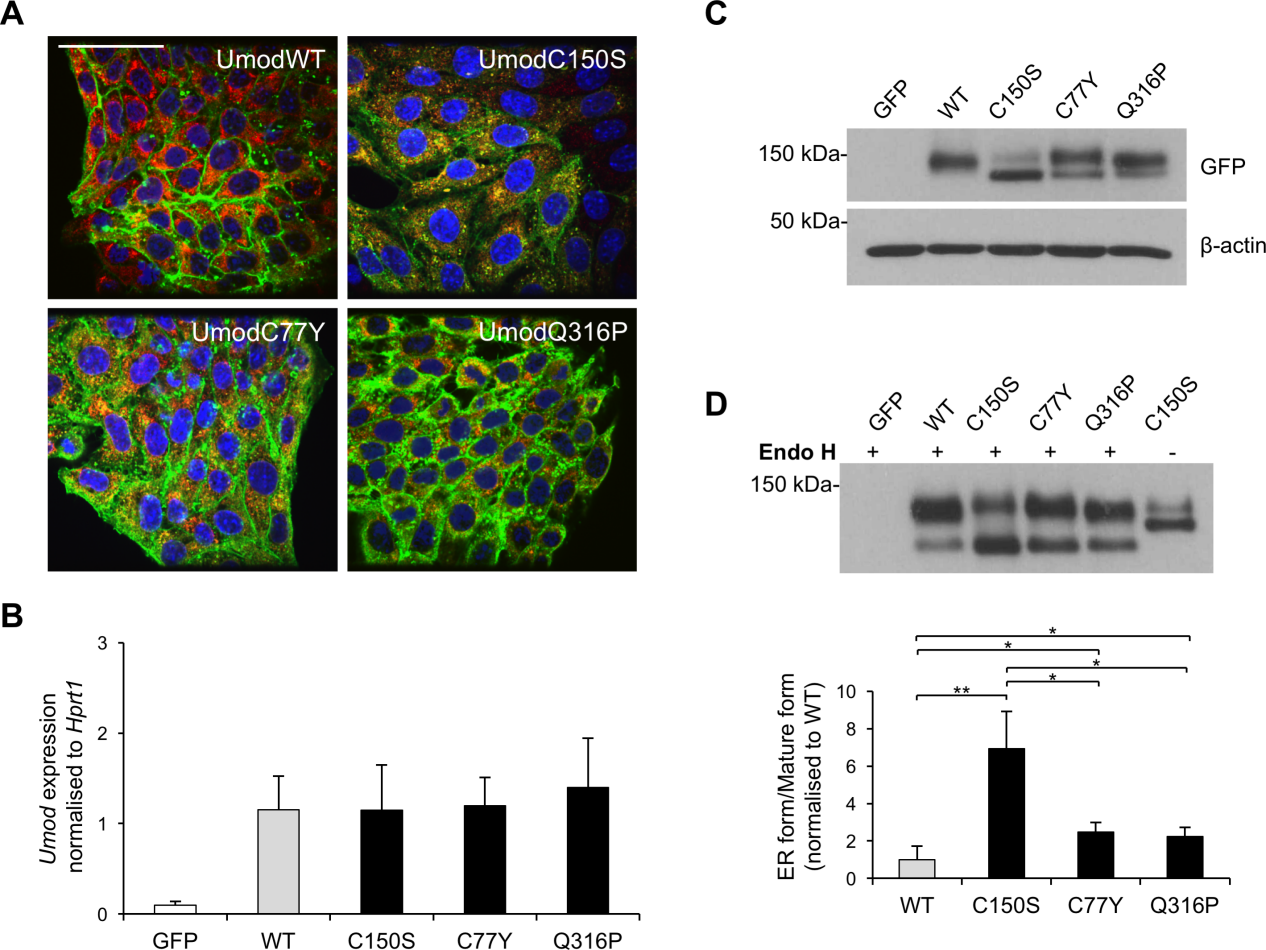
Characterisation of trafficking of different uromodulin mutants in mTAL cells. (A) Immunofluorescence analysis of mTAL cells expressing the indicated uromodulin isoforms. A merge picture of signals obtained for uromodulin (green), calreticulin (ER marker, red) and dapi (blue) is shown. Bar = 40 μm. (B) Uromodulin expression assessed by real time RT-qPCR. Expression is normalised to *Hprt1*. Cells expressing GFP alone are shown as negative control. (n = 6 independent experiments) (C) Western blot analysis of uromodulin expression in lysates of mTAL cells expressing the indicated uromodulin isoforms. β-actin is used as a loading control. (D) Cell lysates were deglycosylated with Endo H and analysed by Western-blot. The quantification of the ratio ER glycosylated form/mature form is shown below. *P<0.05, **P<0.01 (Student t test) (n = 4 independent experiments).

We investigated if our results on UPR activation could be extended to *UMOD* mutations leading to a less severe cellular phenotype, i.e. milder ER retention. Indeed, we previously reported that trafficking defect differs between different mutations [25]. We hence generated new cell lines in mTAL cells expressing uromodulin isoforms with mutations reported in ADTKD-*UMOD* patients [38]. We choose two missense mutations, affecting cysteine (C77Y) [39] and glutamine (Q316P) [40] residues. Both mutations clearly showed increased ER retention, though at a milder extent compared to the C150S mutant, as seen by immunofluorescence (Fig 6A) and biochemistry (Fig 6C and 6D) analyses. The different cell lines are comparable in terms of expression level of mutant uromodulin (Fig 6B).

**Fig 7:**
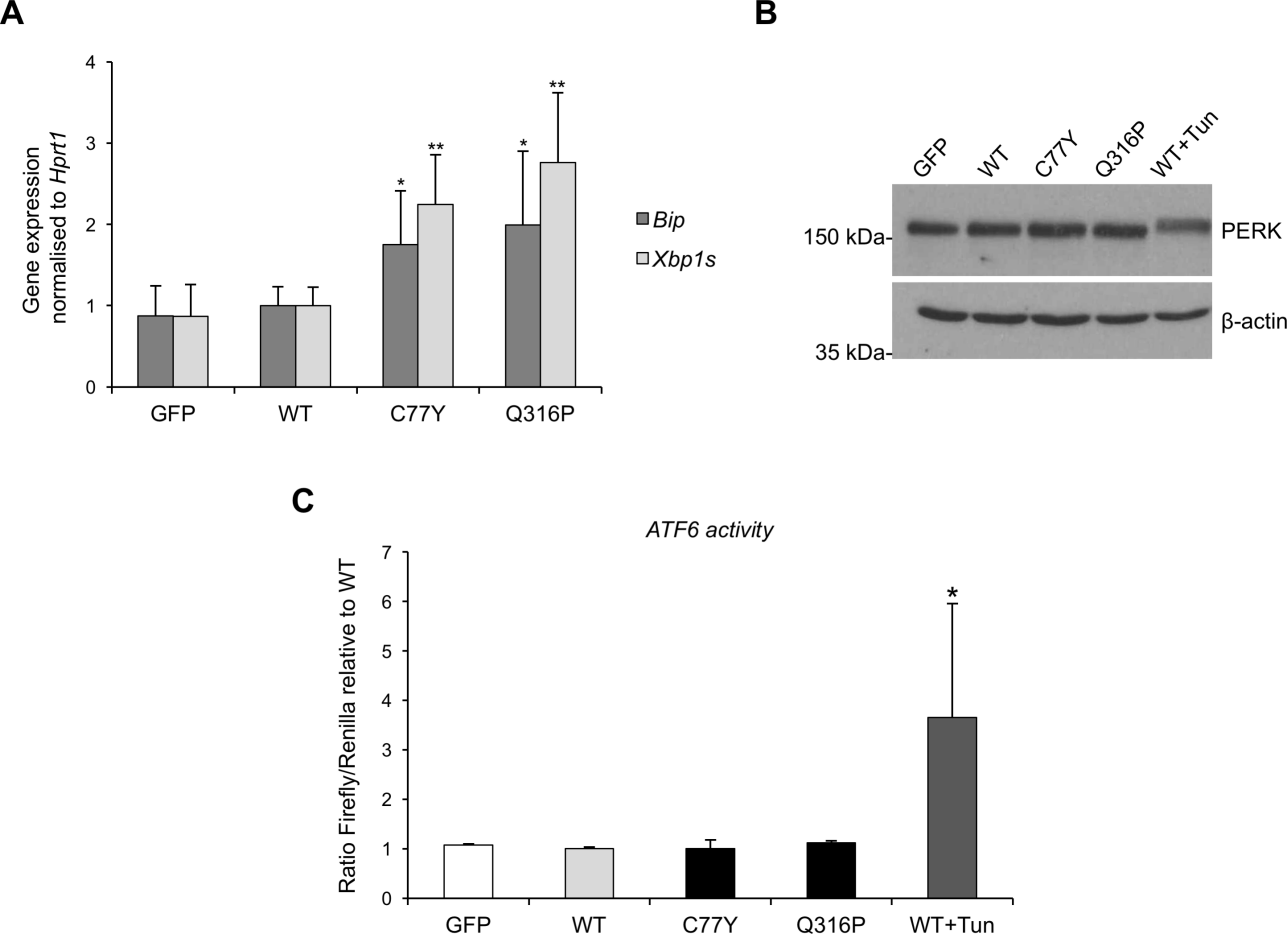
Analysis of UPR induction in mTAL cells expressing different uromodulin mutants. (A) *Bip* and spliced *Xbp1* expression assessed by real time RT-qPCR. Expression is normalised to Hprt1. *P<0.05, **P<0.01 (Student t test *vs* GFP and WT) (n = 6 independent experiments). (B) Western blot analysis of PERK in mTAL cells expressing mutant uromodulin isoforms. None of the mutant isoforms is inducing PERK phosphorylation, seen as a shift in protein migration, as observed upon tunicamycin treatment (2 μg/mL for 14 h). (C) ATF6 activity as assessed by use of an ATF6 reporter construct. No ATF6 activation is observed in mutant uromodulin expressing cells. WT cells treated with tunicamycin are shown as a positive control *P<0.05 (Student t test *vs* WT) (n = 6 independent experiments).

We analysed the three branches of the UPR for these additional mutants. Both mutants lead to ER stress, as seen by the induction of *Bip* expression (Fig 7A). Activation of the IRE1 branch, assessed through the RNA level of spliced *Xbp1*, is observed for both new mutants (Fig 7A), while we did not detect any significant increase of PERK phosphorylation or ATF6 activation (Fig 7B and 7C). These results are comparable to what observed for the C150S mutant.

## Discussion

In this study, we took advantage of newly generated cell models to study the cellular response to mutant uromodulin expression. As previously described [24,25], mutant uromodulin isoforms are largely retained in the ER, likely due to protein misfolding. By metabolic labelling we show that the ER retained protein is degraded via the proteasome pathway, as observed for the C112Y uromodulin mutant [41]. We also demonstrate for the first time that mutant uromodulin shows increased interaction with several ER chaperones, namely calnexin, PDI and BiP, suggesting that uromodulin enters the calnexin cycle to be properly folded [42]. Consistently, our transcriptional profiling study shows that expression of mutant uromodulin mainly impacts on ER function, including protein folding and ER-associated degradation (ERAD) and induces stress response. This is in line with evidence of increased BiP signal in the kidney of ADTKD-*UMOD* patients [43] and mouse models [19]. Our data also reveal that ER stress activates the UPR, a cellular response to perturbed ER homeostasis aimed at diminishing ER load by reducing the amount of protein entering the ER while increasing ER folding capacity and ERAD [31]. In mammalian cells, UPR can be induced by three different sensors: IRE1, PERK and ATF6. An in depth analysis of the UPR in our cellular models shows that mutant uromodulin selectively induces the IRE1 branch of the UPR, as seen by induction of XBP1 splicing, with no activation of the ATF6 and PERK pathways.

The UPR is known to be involved in many physiological processes [44]. In particular, the IRE1-XBP1 pathway is important for the differentiation of professional secretory cells as for example β cells [45]. Moreover, induction of the UPR has been documented in a growing number of diseases [46]. Activation of this stress response greatly impacts on cell fate and function and can promote cell survival or cell death [31]. Our experiments did not show induction of apoptosis, in contrast with previous studies in other cellular models of mutant uromodulin expression [41,47]. However, lack of apoptosis in cells expressing mutant uromodulin is consistent with our previous findings in an *in vivo* model of ADTKD-*UMOD* [19]. Absence of cell death following chronic induction of UPR is suggestive of an adaptive response, although we cannot exclude that *in vivo* UPR may be maladaptive, leading for instance to TAL cell dedifferentiation and loss of function. This dual effect of the UPR in conformational diseases was already reported for neurodegenerative diseases, where the role of spliced Xbp1 seems to differ depending on the context. In some diseases as Huntington’s disease, spliced Xbp1 seems to have a deleterious effect and its absence leads to an improvement of the disease [48]. In other cases, as in Parkinson disease, its induction is beneficial and its overexpression increases the survival of dopaminergic cells [49].

Induction of the UPR has also been detected in renal conformational diseases but its pathogenic role is still unclear and also in this case it differs depending on the disease. Selective activation of UPR is observed in nephrotic syndrome with nephrin mutations [50]. In this context, the PERK branch was not activated, while the ATF6 one was induced by some mutants and considered as adaptive or cytoprotective. Unfortunately, the IRE1 branch was not investigated in this study. The UPR is also induced upon expression of mutant collagen IV, responsible for thin-basement-membrane nephropathy and Alport syndrome [51], collectively referred to as collagen IV nephropathies. In this case activation of the ATF6 branch was not assessed, while both PERK and IRE1 branches were activated with induction of the pro-apoptotic factor Chop, suggesting a maladaptive response. ER stress followed by induction of Chop was also observed upon expression of a mutant isoform of laminin β2 impairing protein secretion and responsible for a mild form of Pierson syndrome [52].

Selective activation of the IRE1 branch of the UPR has already been observed in the kidney following knock out of *Sec63*, a gene encoding a component of the ER translocon complex, a channel through which nascent polypeptides are imported in the ER lumen. Inactivation of *Sec63* in renal distal tubules selectively activates the IRE1 branch of the UPR and leads to renal cyst formation [53]. Inactivating Xbp1 in the *Sec63* knock-out model exacerbates the cystic phenotype while overexpression of spliced Xbp1 ameliorates the phenotype, elegantly demonstrating a protective role of the IRE1 branch of the UPR in this model. A protective role of spliced Xbp1 in the context of *Sec63* deficiency has also been recently shown in podocytes, where it allows the maintenance of a normal glomerular filtration barrier [54]. It is to note that mutations in *Sec61a1*, another component of the translocon, are responsible for ADTKD [5]. In regard of these results targeting the IRE1 pathway could be an interesting therapeutic approach for ADTKD [55].

An important point to take into account is that our study has been performed in a condition of “chronic” expression of uromodulin, where cells have adapted to the high ER load due to uromodulin expression. This allows us to study cellular responses to the chronic accumulation of mutant uromodulin, a condition that is similar to the one *in vivo*. It would also be interesting to study these pathways in models of inducible uromodulin expression to follow the early, acute cell responses. Indeed, it has already been reported that different branches of the UPR can be induced with a different timing [56,57] and that the relative dynamics of the different branches determines the shift from cell survival to death [58]. A thorough knowledge of the dynamics and of the pro-survival or apoptotic significance of UPR induction in ADTKD-*UMOD* would be of interest to develop therapeutic strategies aimed at targeting this stress response. Indeed, it has already been shown for a number of diseases that modulating UPR can be beneficial, but basic knowledge is critical in order to understand how to specifically tune its three branches.

A broader approach, as the use of chemical chaperones that would increase uromodulin trafficking to the plasma membrane and decreased ER load was proposed based on *in vitro* studies showing that 4-phenylbutyrate could be effective [47,59]. However, this strategy had no beneficial effect when tested in mouse models of ADTKD-*UMOD* [21]. Moreover, restoring trafficking of mutant uromodulin should be considered with care, as mutant uromodulin tends to form extracellular aggregates with potential, damaging effect for kidney function [60]. Strategies aimed at modulating the UPR and ERAD may hence be more effective.

This study, by identifying genes up-regulated in cells expressing mutant uromodulin, could also be of interest for the discovery of biomarkers for ADTKD-*UMOD*. Interestingly, we identified the gene encoding mesencephalic astrocyte–derived neurotrophic factor (*Manf*) among the top up-regulated genes in cells expressing mutant uromodulin (Fig 3A). Manf, an ER protein that is secreted during ER stress, has recently been proposed as a urinary diagnostic or prognostic biomarker for ER-stress related kidney diseases [61]. It will hence be interesting to analyse Manf levels in the urine of ADTKD-*UMOD* mouse models and patients to assess its potential use as a marker of disease progression.

Very interestingly, a recent study by Kemter et al. that was published during the revision process of this manuscript, starting from proteome profiling investigated UPR induction in the outer medulla of kidneys of ADTKD-*UMOD* mouse models [62]. The authors observed induction of the PERK and ATF6 branches, while the IRE1 pathway was not investigated. This discrepancy with our findings could be explained by the use of different mutations, or by a diverse response of differentiated TAL cells *in vivo* compared to undifferentiated cell models. Nevertheless, this study confirms and extends our findings *in vivo*, pointing at the UPR as a hallmark of ADTKD-*UMOD* downstream of mutant uromodulin ER retention.

In conclusion, our work demonstrates that mutant uromodulin expression in cell models impacts on ER function and induces the UPR response. This sets the bases for future work to assess the role of the UPR *in vivo* and develop therapeutic strategies aimed at modulating ER stress and homeostasis.

## Materials and methods

### Constructs

The reporter construct for ATF6, p5xATF6-GL3, was created by Ron Prywes [37] and was obtained from Addgene (Addgene plasmid # 11976). The reporter construct for ATF4, ATF4 3: Mouse ATF4 (CHOP11/cATF), 5'UTR and AUG-luc was created by David Ron [34] and obtained from Addgene (Addgene plasmid # 21850).

Human uromodulin expression was induced in mTAL and MDCK cells by transduction with lentiviral particles. The transfer vectors used to clone GFP-tagged wild-type uromodulin were pCCLsin.PPT.SFFV.eGFP.Wpre [63] and pRRLsin.PPTs.hCMV.GFP.Wpre [64] for mTAL and MDCK cells respectively. Transfer vectors for mutant uromodulin isoforms were obtained by mutagenesis using the Quickchange Lightning mutagenesis kit (Stratagene, La Jolla, CA) following the manufacturer’s instructions. Primers were designed using the software QuikChange® Primer Design Program (h*UMOD* C150S Forward 5’- GATGGCACTGTGAGTCCTCCCCGGGCTCCTG-3’; Reverse 5’- CAGGAGCCCGGGGAGGACTCACAGTGCCATC-3’ C77Y Forward 5’-CCTGGAGCTCACAACTACTCCGCCAACAGC-3'; Reverse 5'-GCTGTTGGCGGAGTAGTTGTGAGCTCCAGG-3'; Q316P Forward 5'-GCAGATGGCACTGCCCGTGCAAACAGGACTT-3'; Reverse 5'-AAGTCCTGTTTGCACGGGCAGTGCCATCTGC-3'). Lentiviral particles were produced in HEK293T cells by transient four-plasmid (transfer, packaging (pMDLg/p.RRE and pILV001), and envelope (pMD2.VSV-g)) transfection by calcium phosphate precipitation [65] and concentrated by ultracentrifugation.

### Cell lines

mTAL cells were a generous gift from Prof. S. Bachmann. Cells were derived by microdissection of the TAL segments of the immortomouse® (Charles River, Wilmington, MA) [22]. They were grown in DMEM/F12 (Thermofisher, Waltham, MA) supplemented with 10% FBS (Pan Biotech, Aidenbach, Germany), 100 U/mL penicillin, 100 μg/mL streptomycin (Thermofisher), 5 μg/mL transferrin (Sigma, Saint Louis, MO), 5 ng/mL interferon gamma (Pan Biotech), 4 μg/mL dexamethasone (Sigma), 20 nM selenium (Sigma), 50 μM ascorbic acid (Sigma) and 0,3 g/500 mL NaHCO_3_ (Thermofisher) at 37°C, 5% CO_2_.

MDCK cells [66] were grown in DMEM (Thermofisher) supplemented with 10% fetal bovine serum (Euroclone, Pero, Italy), 200 U/mL penicillin, 200 μg/mL streptomycin and 2 mM glutamine (Thermofisher) at 37°C, 5% CO_2_.

All experiments were performed using mixed populations obtained after transduction with lentiviral constructs expressing the indicated uromodulin isoforms.

### RNA sequencing

RNA was extracted from confluent mTAL cells grown in 35 mm dishes using 1 mL Isol-RNA Lysis Reagent (5Prime, Hilden, Germany) following the manufacturer protocol. Extracted RNA was treated with RNase-free DNase (Qiagen, Venlo, Netherlands) and cleaned-up using the RNeasy Mini kit (Qiagen). RNA-Seq libraries were prepared for each RNA sample using the TruSeq RNA sample prep kit V2 set B (Illumina, San Diego, CA). Each library was sequenced using 101 bp paired-end SBS protocol, four libraries were sequenced on Illumina® HiSeq 2500 sequencer, while two of them were sequenced on Illumina® NextSeq 500, at a final concentration of 8 pM and 1.1 pM, respectively.

After demultiplexing with CASAVA software (version 1.8) the obtained fastq files were aligned on the reference genome using STAR aligner [67]. As reference we used the *Mus musculus* reference genome, build mm10 from the UCSC database adding a “custom chromosome” with the sequence of human *UMOD* gene in order to correctly align those reads obtained from transduced *UMOD*. SAMtools [68] was then used to sort mapped reads by chromosomal coordinate and to convert the file format from SAM to BAM. We added the lentiviral construct features to the standard Ensemble GTF mm10 file as for reference genome. The expression levels were estimated using htseq-count [69] software using the following parameters:—type "exon"—idattr "gene_name"—mode "union". This approach yielded read counts for a total of 38,334 genes, from these we excluded all genes that had less than one count per million in one of the samples, yielding a total of 12,346 genes.

Count data were analysed in R with LIMMA Bioconductor software package [70] and the RNA-Seq-specific function 'voom' to identify differentially expressed genes between wild type and mutant samples. We modelled the contrast matrix to fit the linear model taking into account the two different sequencing batches, in order to remove the source of noise and to obtain correct statistical inference. Statistical analysis was performed by applying the Benjamini–Hochberg correction to calculate the adjusted P-value.

Pathway analysis was performed using Gene Set Enrichment Analysis (GSEA) software (software.broadinstitute.org/gsea) [29,30], using the list of coding genes (identified by their HUGO gene symbol) expressed in mTALs ranked by fold change in C150S relative to wild type. We generated a tailored “GENE_SYMBOL” file, containing the official HUGO symbol for all the coding genes listed in the mTAL cell transcriptome. This allowed the inclusion of all identified genes in the pathway enrichment analysis. Analysis was performed with diverse gene sets including KEGG, Reactome, Biocarta as well as Gene Ontology dataset (biological process, cell component, molecular function). The number of gene set permutations was set to 1,000 and pathways with an FDR value ≤ 0.05 were considered as significantly enriched. Network analysis was performed with the STRING software (string-db.org) [28], using as input all up- or down-regulated genes with an adjusted P<0.05. Protein-protein interaction sources included text mining, experiments, databases, co-expression, neighbourhood and co-occurrence. Search was done on *Mus musculus*.

### Real-time RT-qPCR

RNA was extracted from confluent mTAL or MDCK cells grown in 35 mm dishes using 1 mL Isol-RNA Lysis Reagent (5Prime) following the manufacturer protocol. Extracted RNA was treated with RNase-free DNase (Qiagen) and cleaned-up using the RNeasy Mini kit (Qiagen). RNA was reverse-transcribed using the iScript Reverse Transcription kit (Bio-Rad, Hercules, CA). Real-time RT-qPCR was performed on the LightCycler 480 instrument (Roche, Basel, Switzerland) using the qPCR Core kit for SYBR® Green I No ROX (Eurogentec, Liège, Belgium) with specific primers for the indicated genes (Table 2).

**Table 2:** Real-time RT-qPCR primers.

Target gene	Primer Forward (5’->3’)	Primer Reverse (5’->3’)
*UMOD* (Human)	GCGTACTGCACAGACCCCAGC	GTCATTGAAGCCCGAGCACCG
*Hspa5* (Mouse)	GGGCACGGTGGTCGGCATCG	TTTCCTGACATCTTTGCCCG
*HSPA5* (Dog)	GGTGCCCACCAAGAAGTCTC	GGAGCAGGAGGAATTCCAGT
*Xbp1s* (Mouse)	GAGTCCGCAGCAGGTG	GTGTCAGAGTCCATGGGA
*XBP1S* (Dog)	GAGTCCGCAGCAGGTG	CTGTCAGAATCCATGGGG
*Pdia6* (Mouse)	TAAAGTCGGGGCCGTCAATG	GAGGGCGGCATCTACAATGG
*Pdia4* (Mouse)	GCCGGAGATGCACACGAA	AGCACTGTATCTTTGTCAGCCA
*Manf* (Mouse)	AGTTTTGCCGTGAAGCAAGAG	GCTCAGGTCAATCTGCTTGT
*Dnajc3* (Mouse)	AAGGGAAGCTTGACGAAGCA	TAGCAGCAGTGTAATCGGCA
*Hprt1* (Mouse)	ACATTGTGGCCCTCTGTGTG	TTATGTCCCCCGTTGACTGA
*Hprt1* (Dog)	ACACTGGGAAAACAATGCAGAC	TCAGGTTTATAGCCAACACTTCG

### Western blot

mTAL or MDCK cells were grown in 35 mm dishes in complete medium. Cells were lysed in 300 μl of octylglucoside lysis buffer (50 mM Tris-HCl, pH 7.4, 150 mM NaCl, 60 mM octyl β-D-glucopyranoside, 10 mM NaF, 0.5 mM Sodium orthovanadate, 1 mM glycerophosphate and protease inhibitor cocktail (Sigma)) for 1 h at 4°C under rotation followed by centrifugation 10 min at 17,000 g. Soluble fractions were quantified by the Bio-Rad Protein Assay (Bio-Rad). Twenty to fifty μg of each protein lysate were loaded onto reducing SDS-polyacrylamide gel electrophoresis (PAGE). Transblotted nitrocellulose membranes (GE Healthcare, Little Chalfont, Buckinghamshire, United Kingdom) were incubated with the indicated primary antibody (rabbit polyclonal anti GFP (A11122, Thermofisher, dilution 1:10,000); rabbit monoclonal anti-PERK (#3192, Cell Signaling, Danvers, MA, dilution 1:1,000); rabbit monoclonal anti-phospho-PERK (Thr980) (#3179, Cell Signaling, dilution 1:1,000); goat polyclonal anti-Bip (sc-1051, Santa Cruz, Dallas, TX, dilution 1:1,000); rabbit polyclonal anti-calnexin (C4731, Sigma, dilution 1:15,000), rabbit polyclonal anti-PDI (sc-20132, Santa Cruz, dilution 1:1,000); mouse monoclonal anti-β-actin (A2228, Sigma, dilution 1:20,000)) followed by incubation with the appropriate horseradish peroxidase-conjugated secondary antibody (1:7,500 dilution; GE Healthcare). Protein bands were visualized with the Immobilon Western Chemiluminescent Horseradish Peroxidase Substrate kit (Millipore, Billerica, MA).

### Pulse-chase experiment

mTAL expressing wild-type or C150S mutant uromodulin isoforms were grown in 35 mm dishes in complete medium. Before labelling cells were washed twice with PBS and starved for 30 min at 37°C in DMEM without methionine and cysteine (Thermofisher) supplemented with 2% dialysed FBS. Labelling was performed for 30 min in the starvation medium supplemented with 100 μCi of EasyTag™ EXPRESS35S Protein Labeling Mix (PerkinElmer, Waltham, MA) containing ^35^S-methionine and ^35^S-cysteine. After washing of the labelling medium, chase was performed in complete medium supplemented with 2.5 mM methionine for the indicated time. When indicated chase was performed in presence of 5 μM MG132 or 100 nM bafilomycin. Cells were lysed in 500 μl of octylglucoside lysis buffer (see above). Protein lysates were loaded onto protein G -Sepharose beads (GE Healthcare) pre-conjugated with 6 μl of sheep polyclonal anti-uromodulin antibody (T0850B, United States Biological, Salem, MA) and incubated 16 hr at 4°C. After washes in 50mM Tris-HCl pH 7.4; 150 mM NaCl and IGEPAL 0.5%, beads were resuspended in Laemmli Buffer. Immuno-precipitated proteins were separated on reducing 8% SDS-PAGE and radioactivity was revealed by exposure of Imaging plate (Fujifilm, Tokyo, Japan). Quantification was performed using the gel analysis option of ImageJ software.

### Co-immunoprecipitation

mTAL cells were grown to confluence in 10 cm dishes and lysed in 1 mL of octylglucoside lysis buffer for 1 h at 4°C under rotation followed by centrifugation 10 min at 17,000 g. Cell lysates (1.5 mg) were incubated under rotation for 4 h at 4°C with 20 μl Protein G-Sepharose beads for pre-clearing (GE Healthcare). Pre-cleared lysates were then incubated under rotation for 16 hours at 4°C with 20 μl protein G-Sepharose beads pre-conjugated with 6 μl of sheep polyclonal anti-uromodulin antibody (T0850B, United States Biological) for co-immunoprecipitation of calnexin and PDI or with 6 μl of antiserum to human uromodulin (generous gift from Prof. Serafini-Cessi) [71] for BIP co-immunoprecipitation. Beads were washed 3 times in 50mM Tris-HCl pH 7.4; 150 mM NaCl and IGEPAL 0.5%. Immuno-precipitated material was eluted by incubation 10 min at 98°C in 50 μl of 100 mM Tris-HCl pH 8, 4% SDS.

### Immunofluorescence

Cells grown on coverslip in 12-wells plate were fixed in 4% paraformaldehyde (PFA) for 20 min, permeabilised 10 min with 0.5% triton and blocked 30 min with 10% donkey serum. Cells were labelled for 1 h 30 min at room temperature with the indicated antibodies (goat polyclonal anti-uromodulin (0855140, MP Biomedicals, Santa Ana, CA, dilution 1:500); rabbit polyclonal anti-calreticulin (C4606, Sigma, dilution 1:500); mouse monoclonal anti KDEL (ADI-SPA-827, Enzo Life Sciences, Farmingdale, NY, dilution 1:500) followed by 1h incubation with the appropriate Alexa-Fluor conjugated secondary antibodies (1:500; Thermofisher). Cells were stained with 4,6-diamidino-2-phenylindole (DAPI) and mounted using FluorSave Reagent (Calbiochem, San Diego, CA). All pictures were taken with an UltraVIEW ERS spinning disk confocal microscope (UltraVIEW ERS-Imaging Suite Software, Zeiss 63X/1.4; PerkinElmer Life and Analytical Sciences Boston, MA). All images were imported in Photoshop CS (Adobe Systems, Mountain View, CA) and adjusted for brightness and contrast.

## Supporting information

S1 Fig Pathway analysis in mTAL cells expressing uromodulin. (A) Enrichment plots (GSEA) for Gene Ontology gene sets showing the highest enrichment score for Cell Component (left panel) and Biological Process (right panel) in mutant expressing cells compared to wild type ones. (B) Validation of RNA sequencing data by real-time RT-qPCR. Expression fold change for ER stress-related genes *Pdia6*, *Pdia4*, *Manf* and *Dnajc3* in mutant expressing cells relative to wild type ones, as obtained by RNA sequencing (black) and by real-time RT-qPCR (white). Real-time RT-qPCR results are normalised to *Hprt1*. *P<0.05, **P<0.01 and ***P<0.005 (mutant *vs* wild type, Student t test). (C) Western blot analysis showing absence of expression of cleaved caspase 3 in mTAL cells expressing wild type or C150S uromodulin. Cells treated with staurosporine (1 μM for 4 h) are shown as a positive control.
(TIF)

S2 Fig Characterisation of MDCK cells expressing GFP-tagged wild type or C150S uromodulin isoforms. (A) Live imaging showing GFP signal in MDCK cells expressing wild type or C150S uromodulin. Bar = 40 μm. (B) Uromodulin expression assessed by real-time RT-qPCR. Expression is normalised to *HPRT1*. (n = 5 independent experiments) (C) Western-blot analysis of MDCK cells expressing wild type or C150S uromodulin. * indicates the ER-type glycosylated form of uromodulin that is Endo H sensitive (see panel below). (D) Immunofluorescence analysis of MDCK cells expressing GFP-tagged uromodulin isoforms. GFP signal is shown in green. KDEL, used as an ER marker, is shown in red. Merged pictures show ER localisation of mutant uromodulin isoform while the wild type protein is enriched at the plasma membrane. Bar = 40 μm.
(TIF)
