# Supplementary figures and images for: Mutant uromodulin expression leads to altered homeostasis of the endoplasmic reticulum and activates the unfolded protein response
Source: PLoS One. 2017 Apr 24;12(4):e0175970. doi: 10.1371/journal.pone.0175970 (PMC5402980; doi:10.1371/journal.pone.0175970)

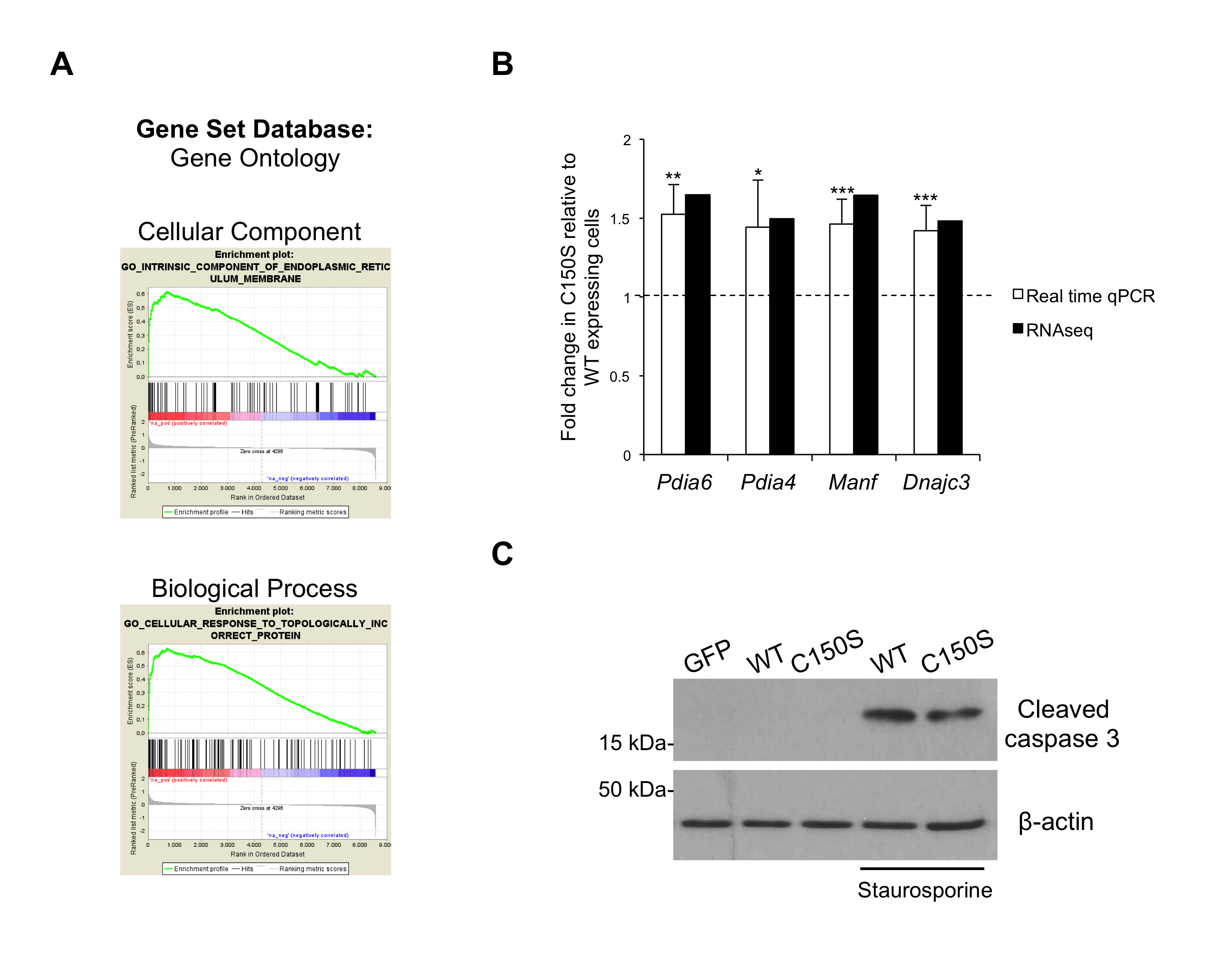

Supplement: S1 Fig — (A) Enrichment plots (GSEA) for Gene Ontology gene sets showing the highest enrichment score for Cell Component (left panel) and Biological Process (right panel) in mutant expressing cells compared to wild type ones. (B) Validation of RNA sequencing data by real-time RT-qPCR. Expression fold change for ER stress-related genes Pdia6, Pdia4, Manf and Dnajc3 in mutant expressing cells relative to wild type ones, as obtained by RNA sequencing (black) and by real-time RT-qPCR (white). Real-time RT-qPCR results are normalised to Hprt1. *P<0.05, **P<0.01 and ***P<0.005 (mutant vs wild type, Student t test). (C) Western blot analysis showing absence of expression of cleaved caspase 3 in mTAL cells expressing wild type or C150S uromodulin. Cells treated with staurosporine (1 μM for 4 h) are shown as a positive control. (TIF) [file pone.0175970.s001.tif]

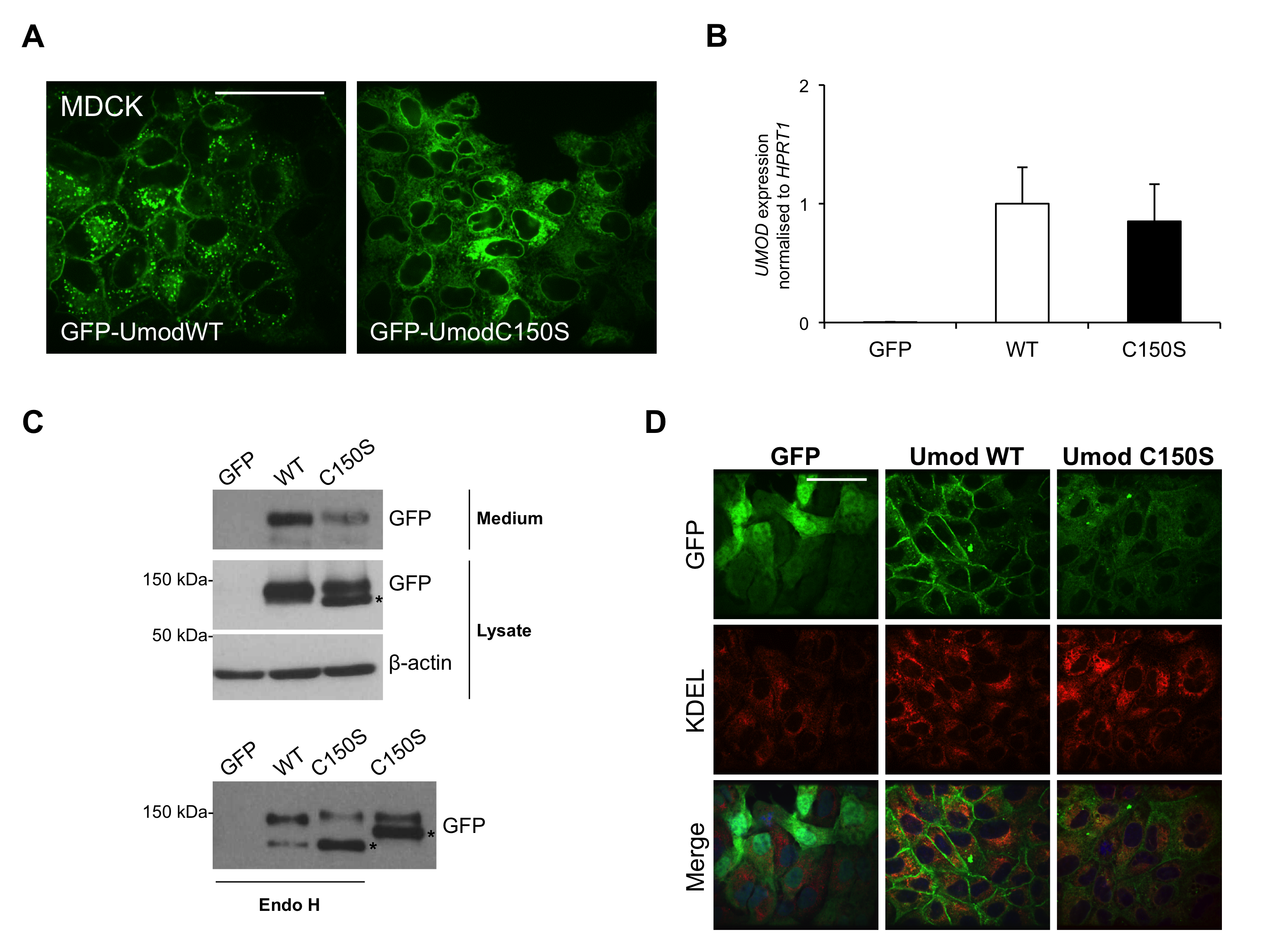

Supplement: S2 Fig — (A) Live imaging showing GFP signal in MDCK cells expressing wild type or C150S uromodulin. Bar = 40 μm. (B) Uromodulin expression assessed by real-time RT-qPCR. Expression is normalised to HPRT1. (n = 5 independent experiments) (C) Western-blot analysis of MDCK cells expressing wild type or C150S uromodulin. * indicates the ER-type glycosylated form of uromodulin that is Endo H sensitive (see panel below). (D) Immunofluorescence analysis of MDCK cells expressing GFP-tagged uromodulin isoforms. GFP signal is shown in green. KDEL, used as an ER marker, is shown in red. Merged pictures show ER localisation of mutant uromodulin isoform while the wild type protein is enriched at the plasma membrane. Bar = 40 μm. (TIF) [file pone.0175970.s002.tif]
